# Book Review

**Published:** 1947

**Authors:** 


### Reviews of Books

**The British Red Cross Society—Quarterly Review :** Vol. 34, No. 1. January 1947.—This review is wide in its scope and full of interest. Articles with a strong appeal deal with the experiments into the nature of the " common cold " now taking place at the Harvard Hospital, Salisbury, and a review of the Convalescent Home and Auxiliary Hospital Scheme devized in 1946 by the British Red Cross Society. The Booklet is of handy size, beautifully printed and well illustrated. It should command popularity in both lay and professional circles.

**Anatomical Terms : their origin and derivation.** By E. J. Field, M.D., M.S., and R. J. Harrison, M.B., B.Ch. Pp. vi, 163. Cambridge : W. Heffer and Sons Ltd. 1947. Price 7/6.—The authors of this little book announce their purpose as first explaining to the non-classical medical student the meaning and derivation of anatomical terms ; and secondly of offering short notes on those anatomists whose names are associated with various structures. It is not easy to appreciate exactly what is to be included and why : for example, " Ductus, (L) a duct ; Longus, (L) long ; Discus, (L) a disc," are surely unnecessary—and the last of Greek rather than Latin ancestry. " Fremitus, Scalpel, Myxoedema, Sphygmograph, Caul " have nothing to do with anatomy, nor Goethe, Wren or da Vinci with anatomical terms. " Attic, Lyra, Prussac, Gowers " and others are omitted, and " Lancisi " hides under " Stria." The non-classical will be puzzled to read on one page " Bulla = vase ", and on the next " Bulla = knob ". " Addison," an unimportant Viscount, gets eleven lines, with a footnote, " Addison, Thomas, after whom Addison's disease is named, was a different person." Is not " chord " (*e.g.* vocal) derived from " guts " via " cat-gut "—the likeness to " a string of sausages " is remote : and when an Alderman damps his ears it is certainly not to induce *vomiting*. There are some puzzling misprints, *e.g.* " Work " for " Word ", and " Bridge " for " Bridle ", appears twice !

**Eye Surgery.** By H. B. Stallard, *M.B.E.*, M.D., F.R.C.S. Pp. xii, 444. Illustrated. Bristol : John Wright & Sons Ltd. 1946. Price 50s.—This book is not a complete compendium of eye operations, but a personal record of those selected by the author, from a wide experience here and abroad, as the most satisfactory in his well-considered judgement. An opening chapter discusses the eye surgeon and his training, the assistant, the operation theatre, its staff, equipment and management, drugs and dressings, pre- and post-operative treatment and nursing. Following a section on anæsthetics, the operations chosen are described with a wealth of detail appropriate to the precise nature of the subject and to the requirements of younger surgeons, post-graduates training in eye surgery, ophthalmic house-surgeons, registrars and clinical assistants. Older surgeons, although they may in some cases vary in their ideas about certain details of technique and procedure, will read with appreciation this clear, sound and well-reasoned exposition of their handicraft. A considerable amount of space is given to the plastic repair of eyelids, and to reconstructive surgery. Here the lessons of war-surgery are effectively incorporated. The text is copiously illustrated by the author's original pencil-drawings, and he has no reason to be diffident about these on the score of artistic merit. They show clearly what he intends to convey, and it is a pleasure to see a book specially illustrated in this way, with comparatively few borrowed blocks apart from those depicting instruments. The author is to be congratulated on a notable achievement, and the publishers, who have made their usual excellent job of the production, on an important addition to their list of medical publications.

**A Synopsis of Anæsthesia.** By J. ALFRED LEE, M.R.C.S., L.R.C.P., M.M.S.A., D.A. Pp. viii, 254. Illustrated. Bristol: John Wright & Sons Ltd. 1947. Price 12s. 6d.—The science of Anæsthesia, having started upon its second centennium, is now undergoing a period of vigorous growth. The increasing variety of methods and of drugs, and the increasing complexity of modern apparatus, require a standard of knowledge and skill which can now be attained only by the specialist in this widening field. The inception in 1935 of a Diploma in Anæsthesia established the subject on a specialist footing, and the diploma is now a prerequisite to any worthwhile appointment in anæsthesia. It is natural, therefore, that a large number of books on anæsthesia should have been published in recent years; and not a few of them are designed to assist the D.A. candidate. This synopsis is one of the best of these, and it will be found of value as a reference book to everyone interested in the subject. It is a masterpiece of compression by a writer who has read widely and who must often have been tempted to expand. Little of importance has been omitted, and the book is well balanced on the whole, though two pages devoted to the anatomy of the rectum seems a little flatulent. Most of the sections are excellently written. That on the autonomic system is particularly valuable. The section on choice of anæsthetic for various operations is a little perfunctory, and the techniques recommended will not all command universal acceptance. The revision of the text shows evidence of haste. Davy's discovery of nitrous oxide is dated 1778, twenty years too early; the boiling point of ethylene is given as *plus* 103 degrees; the xiphisternum is in one place related to the eighth dorsal segment; the conversion factor for reading helium flow on a nitrous oxide flowmeter is incorrect; and the names of Nosworthy, Freedman and Human are misspelt. These are minor blemishes which can be removed in a subsequent edition. The high standard of the book as a whole and its undoubted value to would-be anæsthetists, as well as to those already established, should ensure that subsequent editions will be called for.

